# Effect of comprehensive geriatric assessment–based nursing intervention in elderly patients with Sarcopenia and osteoporosis

**DOI:** 10.12669/pjms.42.7.16753

**Published:** 2026-07

**Authors:** Huizhu Shi, Liang Chen

**Affiliations:** 1 Huizhu Shi Department of Geriatrics, Changxing County People’s Hospital, Changxing, Zhejiang Province 313100, P.R. China; 2 Liang Chen Department of Orthopedics, Changxing County Traditional Chinese Medicine Hospital, Zhejiang Province 313100, P.R. China

**Keywords:** Bone mineral density, Comprehensive Geriatric Assessment, Nursing intervention, Osteoporosis, Sarcopenia

## Abstract

**Objective:**

To investigate the effect of nursing intervention based on the Comprehensive Geriatric Assessment (CGA) process in elderly patients with sarcopenia and osteoporosis.

**Methodology:**

A retrospective analysis included 152 elderly inpatients with sarcopenia and osteoporosis admitted to the Department of Geriatrics of Changxing County People’s Hospital from January 2024 to June 2025. Based on the nursing protocol, the patients were divided into an observation group (n=76, receiving CGA-based nursing intervention) and a control group (n=76, receiving conventional nursing intervention). Both groups received the corresponding interventions for six months. The appendicular skeletal muscle index (ASMI), grip strength, gait speed, bone mineral density (BMD) T-score, Self-Rating Anxiety Scale (SAS) score, Self-Rating Depression Scale (SDS) score, and post-intervention nursing satisfaction were compared between the two groups.

**Results:**

After intervention, the ASMI, grip strength, gait speed, and BMD T-score in the observation group were significantly higher than those in the control group, while the SAS and SDS scores were significantly lower (all *P*<0.05). The nursing satisfaction rate in the observation group was 94.7%, which was significantly higher than the 84.2% in the control group (*P*<0.05).

**Conclusion:**

CGA-based nursing intervention was demonstrated to be associated with improved muscle function and bone mineral density, alleviated anxiety and depression, and enhanced nursing satisfaction in elderly patients with sarcopenia and osteoporosis.

## INTRODUCTION

Sarcopenia and osteoporosis, two highly prevalent degenerative diseases in the elderly population, are often interrelated and progress synergistically, severely impairing patients’ quality of life.^1^ Sarcopenia is characterized by the loss of skeletal muscle mass, decline in muscle strength, and impairment of muscle function, and is caused by various factors such as aging, nutritional imbalance, and insufficient physical activity.^2^ Osteoporosis, on the other hand, is manifested by decreased bone mass and damaged bone microstructure.^3^ Accumulating evidence suggests that sarcopenia and osteoporosis share overlapping pathophysiological mechanisms, such as chronic low-grade inflammation, endocrine disturbances, and compromised nutrient metabolism. The progression of either disorder may further exacerbate the other, resulting in a self-perpetuating pathological cycle.^1^,^2^,^4^ Therefore, there is a pressing need for developing scientifically and systematically designed intervention measures to improve muscle and bone function simultaneously, thereby slowing disease progression and reducing the incidence of adverse outcomes in elderly patients with sarcopenia and osteoporosis.

Traditional nursing strategies for elderly patients with sarcopenia and osteoporosis mainly focus on medication instruction and routine care, but frequently lack individualized targeting and comprehensive planning, and do not take into account psychological, nutritional, and functional dimensions, which may limit their overall effectiveness.^5^,^6^ Comprehensive Geriatric Assessment (CGA), a systematic tool that enables a comprehensive evaluation of elderly patients across multiple dimensions, including physical function, nutritional status, psychological state, and social support, has been widely applied in the management of elderly patients with chronic diseases.^6^,^7^ However, studies investigating CGA in elderly patients with sarcopenia and osteoporosis remain limited. To fill the gap, this study aimed to construct a CGA-based standardized nursing intervention protocol that includes nutritional support, exercise training, psychological intervention and health management through dynamic assessment and adjustment, and to investigate its effects on elderly patients with sarcopenia and osteoporosis.

## METHODOLOGY

This retrospective analysis included medical records of 152 elderly inpatients with sarcopenia and osteoporosis who were admitted to the Department of Geriatrics of Changxing County People’s Hospital from January 2024 to June 2025. Based on the nursing interventions received, patients were divided into an observation group (n=76, receiving a CGA-based nursing protocol) and a control group (n=76, receiving conventional nursing intervention).

### Ethical approval:

The ethics committee of our hospital approved this study (No.2026-EC-14, Date: March 10, 2026).

### Inclusion Criteria:

- Patients aged 60 to 80.
- Patients met the AWGS 2019 diagnostic criteria for sarcopenia:² Appendicular Skeletal Muscle Index (ASMI) < 7.0 kg/m² in males and < 5.7 kg/m² in females, accompanied by decreased grip strength (males < 28 kg, females < 18 kg) or decreased gait speed (< 1.0 m/s).
- Patients met the diagnostic criteria for osteoporosis:^8^ Bone mineral density (BMD) T-score ≤ -2.5 in the lumbar spine (L1~L4) or femoral neck detected by dual-energy X-ray absorptiometry.
- Clear consciousness and ability to cooperate with assessments and interventions.
- Complete clinical data.

### Exclusion Criteria:

- Complicated with severe dysfunction or failure of important organs such as heart, liver, kidney, and brain.
- Complicated with malignant tumors, infectious diseases, or metabolic diseases (except osteoporosis).
- Presence of limb dysfunction or cognitive impairment, unable to perform exercise training.
- Recent use of drugs affecting muscle function and bone metabolism (such as glucocorticoids and antiepileptic drugs) that cannot be discontinued.

### Nursing Protocols:

The intervention period for both groups was six months, and the specific intervention measures are shown in Table-I.

**Table-I:** Specific intervention measures of the two groups.

Intervention Dimension	Control Group (Traditional Nursing Intervention)	Observation Group (CGA-Based Nursing Intervention)
Team Configuration	Implemented solely by the responsible nurse, with no multidisciplinary collaboration	A multidisciplinary team was established, including geriatricians, responsible nurses, rehabilitation therapists, nutritionists, and psychological counselors, with clear responsibilities for collaborative work
Assessment Phase	No systematic assessment process; only basic disease understanding via medical records	A comprehensive CGA assessment was conducted in the first week of intervention, covering physical function, nutritional status, psychological state, social support, medical history, and medication use. A phased assessment was conducted monthly, with dynamic adjustments to the plan
Medication Management	Medication administration in accordance with the doctor’s instructions; the patient was informed of the usage, precautions, and potential adverse reactions, and was supervised to ensure timely medication	Based on the control group’s approach, medication efficacy and adverse reactions were regularly evaluated, medication guidance was optimized based on phased assessment results, and the medication safety and compliance were ensured
Nutritional Intervention	No personalized nutrition plan; only oral advice on a rational diet	A nutritionist developed a personalized nutrition plan based on the MNA scale and nutritional indicators, ensuring daily protein intake (1.2–1.5 g/kg), calcium (1000–1200 mg), and vitamin D (800 IU) intake. Nutritional supplements were provided if necessary for patients with feeding difficulties
Exercise Intervention	Simple guidance on low-intensity activities (e.g., walking, tai chi) with no unified plan, time, or intensity requirements	A rehabilitation therapist developed a graded exercise program, including resistance training (elastic band exercise, 3 times/week × 30 min), balance training (single-leg standing, tai chi, 3 times/week × 20 min), and aerobic exercise (walking/jogging, 5 times/week × 30–40 min). Full-time supervision is provided to prevent falls, with dynamic intensity adjustments
Psychological Intervention	Simple comfort and counseling were provided when negative emotions occurred, with no standardized process	A psychological counselor provided monthly, personalized psychological counseling (cognitive-behavioral therapy and relaxation training). The responsible nurse communicates daily, encourages family emotional support, and builds a family support system
Health Management & Follow-Up	Oral health education + distribution of manual; no regular follow-up mechanism	The responsible nurse conducted 1 follow-up visit per month (in-person/phone/home visit), guides home rehabilitation training, reminds patients of regular re-examinations, and provides health education on disease management and fall prevention
Fall Prevention	Education on fall prevention knowledge was provided; no targeted protective measures	Fall risk assessed and targeted measures implemented for high-risk patients, such as installing home handrails, providing non-slip footwear, and avoiding unsupervised activities. Regular safety inspections of the home environment are also required.

### Observation Indicators:

The following information was collected for all patients:

- Baseline characteristics, including gender, age, BMI, education level, marital status, history of smoking, history of drinking, Charlson Comorbidity Index, types of medications, and caregiver information.
- Muscle function indicators, including ASMI, grip strength, and gait speed. ASMI was measured using bioelectrical impedance analysis, calculated as the ratio of appendicular skeletal muscle mass to height squared. Grip strength was measured with an electronic dynamometer (CSTF-WL, Tsinghua Tongfang, Beijing, China). The patient sat with the arm naturally hanging down, squeezed the dynamometer with maximum force, and the average of three consecutive measurements was taken. Gait speed was measured using the 6-meter walking test. The patient walked six meters at their usual pace, time was recorded, and gait speed (m/s) was calculated.
- Bone density indicators: BMD of the lumbar spine (L1–L4) and femoral neck was measured using dual-energy X-ray absorptiometry (DXA) (GE Lunar DPX Bravo, GE Healthcare Inc., Waukesha, WI, USA), and the BMD T-score was recorded.
- Psychological status indicators. Anxiety and depression are assessed using the Self-Rating Anxiety Scale (SAS) and Self-Rating Depression Scale (SDS). Both scales contain 20 items scored on a 4-point scale, with higher total scores indicating more severe anxiety or depression. A total SAS score ≥ 50 indicates the presence of anxiety, and a total SDS score ≥ 53 indicates the presence of depression.
- Nursing satisfaction was assessed using a self-designed nursing satisfaction questionnaire from our hospital. The questionnaire includes 10 items: service efficiency (waiting time for services), service attitude, service professionalism, service environment, health education and guidance, communication (completeness of the provided information), confidentiality, way questions and queries dealt by staff, care coordination, and overall experience. Each item scored on a 5-point scale, with a total score of 50. The score was categorized as: Very satisfied: 45–50 points; Generally satisfied: 30–44 points; Dissatisfied: < 30 points. Satisfaction rate = (number of very satisfied cases + number of generally satisfied cases) / total number of cases × 100%.

### Statistical Analysis:

All data analyses were performed using SPSS 25.0 (IBM Corp., Armonk, NY, USA). Measurement data were expressed as mean ± standard deviation. Paired t-tests were used to compare within-group changes before and after the intervention. Independent-samples t-tests were used to compare differences between groups. Count data were expressed as the number of cases (percentage) [n (%)], and Chi-square test was used for comparison between groups. A P value < 0.05 was considered statistically significant.

## RESULTS

In this study, a total of 152 elderly patients with sarcopenia and osteoporosis were enrolled, including 57 males and 95 females, aged 62 to 80 years, with a mean age of 73.1 ± 4.6 years. As shown in Table-II, there were no statistically significant differences in baseline characteristics between the two groups (P > 0.05).

**Table-II:** Comparison of baseline characteristics between two groups of patients.

Variables	Observation group (n=76)	Control group (n=76)	χ^2^/t	P
Gender			0.253	0.615
Male	30 (39.5)	27 (35.5)		
Female	46 (60.5)	49 (64.5)		
Age (years)	72.8±4.8	73.5±4.5	-0.857	0.393
BMI (kg/m²)	20.1±1.6	19.9±1.8	0.817	0.415
Education level			2.149	0.342
Primary school	47 (61.8)	46 (60.5)		
Junior high school	16 (21.1)	22 (28.9)		
High school and above	13 (17.1)	8 (10.5)		
Marital status			0.424	0.515
Single/Widowed	37 (48.7)	33 (43.4)		
Married	39 (51.3)	43 (56.6)		
History of Smoking	33 (43.4)	31 (40.8)	0.108	0.742
History of Drinking	32 (42.1)	36 (47.4)	0.426	0.514
Charlson comorbidity index	6.84±1.40	7.04±1.50	-0.84	0.402
Type of medication	5.79±1.35	6.00±1.50	-0.911	0.364
Caregiver			4.108	0.250
Oneself	29 (38.2)	35 (46.1)		
Spouse	26 (34.2)	19 (25.0)		
Children	17 (22.4)	13 (17.1)		
Other	4 (5.3)	9 (11.8)		

Before intervention, there were no statistically significant differences in ASMI, grip strength, gait speed, BMD, SAS score, or SDS score between the two groups (P>0.05). After intervention, the ASMI, grip strength, gait speed, and BMD of patients in both groups were significantly higher than those before the intervention, and markedly higher in the observation group (P<0.05). The post-intervention SAS and SDS scores of both groups significantly decreased, and the scores in the observation group were considerably lower than those in the control group (P<0.05) (Table-III). As shown in Table-IV, after intervention, the nursing satisfaction rate of the observation group was 94.7%, significantly higher than that of the control group (84.2%) (P<0.05).

**Table-III:** Comparisons of ASMI, grip strength, gait speed, bone mineral density, SAS and SDS scores between the two groups.

Variables	Observation group (n=76)	Control group (n=76)	t	P
***Before intervention***				
ASMI	5.66±0.7	5.55±0.72	0.892	0.374
Grip strength	17.7±3.1	17.3±2.9	0.901	0.369
Gait speed	0.43±0.1	0.44±0.12	-0.893	0.373
BMD	-2.95±0.2	-2.99±0.19	1.335	0.184
SAS	53.6±5.4	54.8±5.9	-1.341	0.182
SDS	53.4±4.9	52.1±5.5	1.611	0.109
***After intervention***				
ASMI	6.47±0.867	6.12±0.73	2.692	0.008
Grip strength	21.2±3.4	19.4±3.6	3.243	0.001
Gait speed	0.59±0.13	0.54±0.14	2.298	0.023
BMD	-2.71±0.2	-2.88±0.22	5.089	<0.001
SAS	40.1±4.6	45.9±5.1	-7.377	<0.001
SDS	38.1±6.2	43.4±5.5	-5.540	<0.001

**Table-IV:** Post-intervention nursing satisfaction rate.

Groups	Very satisfied	Generally satisfied	Dissatisfied	Nursing satisfaction rate
Observation group (n=76)	51 (67.1)	21 (27.6)	4 (5.3)	72 (94.7)
Control group (n=76)	41 (53.9)	23 (30.3)	12 (15.8)	64 (84.2)
*χ^2^*				4.471
*P*				0.034

## DISCUSSION

The results of this study demonstrated that CGA-based nursing intervention could significantly improve muscle function and BMD, alleviate anxiety and depression, and enhance nursing satisfaction in elderly patients with sarcopenia and osteoporosis compared to traditional nursing intervention.

The decline in muscle function is a core characteristic of geriatric sarcopenia, which directly impairs patients’ limb mobility and activities of daily living.^9^ The findings of this study showed that the ASMI, grip strength, and gait speed in the observation group were significantly higher than those in the control group after intervention, indicating that CGA-based nursing intervention has a distinct advantage in improving patients’ muscle function. The exercise guidance in traditional nursing intervention lacks individualization and standardization, leading to poor patient compliance and failure to achieve the desired effect of muscle training.^10^ In contrast, CGA-based nursing intervention involves a comprehensive pre-intervention assessment of patients’ muscle function, exercise tolerance, and limb mobility. The results of the assessment are then used to develop graded exercise programs combining resistance, balance, and aerobic exercise to target improvements in muscle mass, strength, and motor function.^11^ A study by Zhou et al.^11^ indicated that resistance training for patients with osteoporosis and sarcopenia improves quadriceps mass, promotes recovery of muscle strength, restores knee joint function, and, to a certain extent, promotes recovery of musculoskeletal system coordination. In this study, dynamic assessments were conducted monthly during the intervention, and exercise programs were adjusted according to patients’ tolerance and training effects to avoid injuries from excessively high exercise intensity or compromised efficacy from insufficient intensity.

This approach significantly enhanced the scientific rigor and effectiveness of the exercise intervention, consistent with the findings of Zhang et al.,^12^ further confirming the role of CGA-based nursing intervention in improving muscle function in elderly patients with sarcopenia. In addition, nutritional supplementation in the observation group in this study provided a material basis for muscle repair and synthesis, thereby promoting skeletal muscle protein synthesis and restoring muscle strength.^13^

Decreased BMD is the main pathological change of osteoporosis. Therefore, increasing BMD is important for slowing the progression of osteoporosis and reducing the risk of fragility fractures.^14^ The results of this study showed that the BMD T-scores in the observation group were significantly higher than those in the control group after intervention, suggesting that CGA-based nursing intervention can effectively improve the BMD of the patients. It is plausible that nursing interventions based on comprehensive geriatric assessment may regulate bone metabolism through a multidimensional approach, incorporating nutritional optimization, structured exercise programs, and individualized medication management.^15^ Calcium and vitamin D supplementation can directly promote bone formation, while protein intake provides raw materials for bone matrix synthesis and improves bone microstructure.^16^ Resistance training and weight-bearing exercise can stimulate bone tissue, enhance osteoblast activity, increase bone mass, improve bone mechanical properties, and slow the decline in BMD.^17^,^18^ A study by Zhang et al.^19^ showed that CGA-based nursing intervention for elderly patients with Type-2 diabetes mellitus complicated by osteoporosis can increase their BMD T-scores. Furthermore, given the synergistic relationship between sarcopenia and osteoporosis, improvements in muscle function may enhance bone formation through mechanical loading and stimulation, while increased BMD provides structural support for muscle activity. Together, these effects contribute to improved musculoskeletal health in affected patients.^16^–^19^

Elderly patients with sarcopenia and osteoporosis are prone to anxiety and depression due to decreased limb function, impaired activities of daily living, and fear of falls and fractures.^20^ The results of this study showed that the SAS and SDS scores in the observation group were significantly lower than those in the control group after intervention, indicating that CGA-based nursing intervention can more effectively alleviate patients’ anxiety and depression. Through pre-intervention assessment of psychological status, CGA–based nursing interventions are able to identify the underlying causes and severity of patients’ anxiety and depression, enabling the development of individualized intervention programs tailored to each patient’s specific needs. Cognitive behavioral therapy, which is adopted to help patients correct erroneous cognitions and relieve psychological pressure, and relaxation training are used to assist patients in regulating emotions and reducing symptoms of anxiety and depression.^20^,^21^ Furthermore, with improvements in muscle function and BMD, patients’ limb mobility and activities of daily living are enhanced, reducing the risk of falls and significantly increasing their self-confidence.^22^ This is consistent with the findings of Jia J et al.,^22^ confirming that a CGA-based psychological intervention combined with physical intervention can significantly improve the mental health of elderly patients.

The results of this study showed that the nursing satisfaction rate of the observation group was significantly higher than that of the control group. These results confirm that CGA-guided, patient-centered nursing allows to develop individualized care strategies tailored to patients’ nutritional, functional, and psychological needs. Combined with health education and home rehabilitation support, this model enhances self-management capacity and continuity of care, ultimately improving patient experience and satisfaction.^23^

### Strength of this study:

The strength of this study lies in the detailed description of the methodology, especially the detailed nursing protocols of the groups, which ensures the reproducibility of the study.

### Limitations

It is a single-center retrospective analysis that included only inpatients aged 60 to 80 years, limiting the generalizability of the results. The intervention period was relatively short (six months in this study), so only short-term intervention effects could be observed, and the impact of long-term intervention on patients’ muscle function, BMD, and prognosis could not be clarified. Biochemical indicators related to muscle function and bone metabolism (e.g., creatine kinase, osteocalcin) were not detected, so the intervention mechanism could not be explored at the molecular level. The impact of differences in patient adherence on intervention effects was not considered. It is suggested that future studies should include more participants, extend the follow-up period, adopt more comprehensive indicators, while also addressing differences in adherence.

## CONCLUSION

CGA-based nursing intervention was demonstrated to be associated with improved muscle function and BMD, alleviated anxiety and depression, and enhanced nursing satisfaction in elderly patients with sarcopenia and osteoporosis. CGA-based nursing intervention, thus, may be considered a scientific and effective nursing model. However, subsequent studies should address the limitations of this study and conduct higher-quality research to provide a more solid evidence-based basis for the nursing management of elderly patients with sarcopenia and osteoporosis.
